# Recognition of TLR2 N-Glycans: Critical Role in ArtinM Immunomodulatory Activity

**DOI:** 10.1371/journal.pone.0098512

**Published:** 2014-06-03

**Authors:** Vania Sammartino Mariano, Andre Luiz Zorzetto-Fernandes, Thiago Aparecido da Silva, Luciana Pereira Ruas, Lilian L. Nohara, Igor Correia de Almeida, Maria Cristina Roque-Barreira

**Affiliations:** 1 Departamento de Biologia Celular e Molecular e Bioagentes Patogênicos, Faculdade de Medicina de Ribeirão Preto, USP, São Paulo, Brasil; 2 Border Biomedical Research Center, Department of Biological Sciences, University of Texas at El Paso, Texas, United States of America; Federal University of São Paulo, Brazil

## Abstract

TLR2 plays a critical role in the protection against *Paracoccidioides brasiliensis* conferred by ArtinM administration. ArtinM, a D-mannose-binding lectin from *Artocarpus heterophyllus*, induces IL-12 production in macrophages and dendritic cells, which accounts for the T helper1 immunity that results from ArtinM administration. We examined the direct interaction of ArtinM with TLR2using HEK293A cells transfected with TLR2, alone or in combination with TLR1 or TLR6, together with accessory proteins. Stimulation with ArtinM induced NF-κB activation and interleukin (IL)-8 production in cells transfected with TLR2, TLR2/1, or TLR2/6. Murine macrophages that were stimulated with ArtinM had augmented TLR2 mRNA expression. Furthermore, pre-incubation of unstimulated macrophages with an anti-TLR2 antibody reduced the cell labeling with ArtinM. In addition, a microplate assay revealed that ArtinM bound to TLR2 molecules that had been captured by specific antibodies from a macrophages lysate. Notably,ArtinM binding to TLR2 was selectively inhibited when the lectin was pre-incubated with mannotriose. The biological relevance of the direct interaction of ArtinM with TLR2 glycans was assessed using macrophages from TLR2-KOmice, which produced significantly lower levels of IL-12 and IL-10 in response to ArtinM than macrophages from wild-type mice. Pre-treatment of murine macrophages with pharmacological inhibitors of signaling molecules demonstrated the involvement of p38 MAPK and JNK in the IL-12 production induced by ArtinM and the involvement ofPI3K in IL-10 production. Thus, ArtinM interacts directly with TLR2 or TLR2 heterodimers in a carbohydrate recognition-dependent manner and functions as a TLR2 agonist with immunomodulatory properties.

## Introduction

The innate immunity is responsible for the recognition and elimination of most microbes. The innate immune response is initiated when conserved molecules on a microorganism called pathogen-associated molecular patterns (PAMPs) activate pattern-recognition receptors (PRRs) on immune cells. Several classes of PRRs have been described. Toll-like receptors (TLRs) are the best-characterized PRRs. The activation of TLRs is important not only for the induction of innate immunity but also for the development of the adaptive response [1]. TLRs are type-I transmembrane glycoproteins, whose solenoid ectodomain interacts with PAMPs. TLRs also have a single transmembrane domain and an intracytoplasmic Toll/IL-1 receptor (TIR) domain. The TIR domain, which is conserved among all TLRs, activates a signaling pathway shared by IL-1R family members. TLRs recruit several adaptor proteins, such as MyD88, Mal, TRAM, and TRIF, through specific TIR-TIR interactions [1], [2]. Subsequently, the transcription factor nuclear factor kappa B (NF-κB) translocates to the nucleus, which results in the induction of a proinflammatory response [3]. Toll-like receptors are constitutively expressed on monocytes, macrophages, and dendritic cells (DCs); certain TLRs are also expressed on other cell types, such as neutrophils, mast cells, epithelial cells, and B cells [4].

TLR2 dimerization with other members of the TLR family, including TLR1 and TLR6, enables TLR2 to recognize different microbial components and to discriminate subtle differences between agonists. TLR2 and TLR6 heterodimers recognize diacylated lipopeptides, such as macrophage-activating lipopeptide 2 (MALP-2) [5]. TLR2 and TLR1heterodimers, in turn, interact with triacylated lipopeptides, such as the synthetic agonist Pam3CSK4 [6]. TLR2/1 and TLR2/6 complexes have been linked to proinflammatory and anti-inflammatory responses, respectively [7], [8]. TLR2 recognition of agonists is also influenced by co-receptors and accessory molecules, such as CD14 [9], CD36 [10], MD-2 [11], lipopolysaccharide-binding protein (LBP) [12], CD11b-CD18 integrin [13], and ganglioside GD1a [14].

ArtinM, a D-mannose-binding lectin obtained from the seeds of *Artocarpus heterophyllus*, is a homotetramer formed by 16-kDa non-glycosylated subunits [15]. Each polypeptide chain contains a carbohydrate recognition domain (CRD) with affinity for Manα1-3[Manα1-6]Man, which corresponds to the core of *N*-linked oligosaccharides. It is well established that ArtinM exerts pleiotropic activities on immune cells, such as neutrophils [15]–[17], mast cells [18], lymphocytes (da Silva et al., in press), macrophages, and dendritic cells [19]. The ArtinM interaction with *N*-linked glycans on the surface of macrophages and dendritic cells, which results in high levels of IL-12 production, is considered a central immunomodulatory event because it drives immunity towards the T helper (Th) 1 axis [20], [21]. This ability accounts for the protection conferred by ArtinM against *Paracoccidioides brasiliensis*, which is dependent on TLR2 [19], [22]. ArtinM also promotes resistance to infection by other intracellular pathogens, such as *Leishmania major* [20], *Leishmania amazonensis* [21], *Neospora caninum* [23], and *Candida albicans* [24].

TLR2 is crucially implicated in the protection against *P. brasiliensis* conferred by ArtinM. Furthermore, TLR2 has N-linked oligosaccharides that are potential targets for ArtinM recognition, and other lectins have been identified as TLR agonists [25]. In the present study, we investigate the direct interaction between TLR2 and ArtinM and the dependence on carbohydrate recognition in that process.

## Materials and Methods

### Ethics statement

The animal studies (protocol number 088/2010) were approved by the Ethical Committee of Ethics in Animal Research (CETEA) of the College of Medicine of Ribeirão Preto of the University of São Paulo and were conducted in accordance with the Ethical Principles in Animal Research adopted by the Brazilian College of Animal Experimentation (COBEA).

### Animals

Male BALB/c, C57BL/6, and TLR2-KO (C57BL/6 genetic background) mice were acquired from the vivarium of the Campus of Ribeirão Preto, University of São Paulo, Ribeirão Preto, São Paulo, Brazil. Animals were housed in the animal facility of the Molecular and Cellular Biology Department of the Faculty of Medicine of Ribeirão Preto, University of São Paulo. All experiments were conducted in accordance with the ethical guidelines of the Animal Studies Ethics Committee of USP-Ribeirão Preto. Mice were used at 6–8 weeks of age.

### ArtinM

ArtinM was purified as previously described [15] from the saline extract of *A. heterophyllus* (jackfruit) seeds through affinity chromatography on sugar columns. Before use, ArtinM aliquots were incubated for 1 h with polymyxin B solution (50 µg/mL) (Sigma-Aldrich, St. Louis, MO) to neutralize any potential contamination with bacterial lipopolysaccharides (LPS).

### Prediction of N-glycosylation sites in TLR2 and TLR4

The amino acid sequences of human and murine TLR2 and TLR4 were obtained from a protein database (http://www.ncbi.nlm.nih.gov/protein) and checked for potential N-glycosylation sites using the NetNGlyc1.0 server (http://www.cbs.dtu.dk/services/NetNGlyc/). Potential sites of N-glycosylation in human and murine TLR2 and TLR4 were identified as sites with a value greater than the default threshold (0.5).

### Expression constructs

Mouse CD14 [26], MD-2 [27], [28], and hemagglutinin (HA) epitope-tagged TLR1, TLR4, TLR6 [29], and TLR2 [26] constructs as well as the β-actin *Renilla* luciferase [30] and the endothelial leukocyte molecule (ELAM)-1-firefly luciferase reporter [31] constructs were kindly provided by Dr. Richard Darveau (University of Washington, Seattle, WA). The mouse CD36 construct [32] was generously provided by Dr. Kathryn J. Moore (Harvard Medical School, Boston, MA). All plasmids used in mammalian cell transfections were purified using an EndoFree Plasmid Purification Maxi Kit (Qiagen) according to manufacturer's instructions.

### HEK293A-cell transfection and luciferase reporter assay for NF-κB activation

Human embryonic kidney (HEK293A) cells, kindly provided by Dr. German Rosas Acosta (University of Texas at El Paso), were cultured in high-glucose Dulbecco's modified Eagle medium (DMEM), supplemented with 10% fetal bovine serum (FBS), at 37°C under a 5% CO_2_ atmosphere. Cell cultures were regularly tested for potential *Mycoplasma* contamination by polymerase-chain reaction [33].

HEK293A cells were seeded on 12-well plates (5×10^5^ cells/well) the day before transfection. The cells were transiently cotransfected with CD14, CD36, MD-2, and a combination of TLR1 and TLR2 (TLR1/2) or TLR2 and TLR6 (TLR2/6) constructs, using Lipofectamine 2000 according to the manufacturer's recommendations. The amount of transfected DNA/well was normalized to 2 µg by adding empty vector. Subsequently, the cells were plated on 96-well plates (4×10^4^ cells/well) at 37°C in DMEM containing 10% FBS. After 24 h, the cells were stimulated with TLR ligands or ArtinM (as indicated in the figures) for 4 h in the luciferase reporter assay or for 20 h for IL-8 detection (described below). For luciferase reporter assays, the cells were also co-transfected with the NF-*κ*B-dependent promoter (ELAM-1-firefly luciferase) and a Renilla luciferase reporter construct (β-actin-Renilla luciferase), which was used to control for transfection efficiency. Then, the HEK293A cells were washed once in PBS and lysed using the Passive Lysis Buffer (Promega). The luciferase activity was measured using the Dual-Luciferase Reporter Assay System (Promega) according to the manufacturer's instructions. The relative luminescence units (RLU) were quantified using the Luminoskan Ascent luminometer (Thermo Fisher Scientific). The luciferase activity was reported as the ratio of NF-κB dependent firefly luciferase activity to constitutively expressed *Renilla* luciferase activity (Luc:Ren luc ratio) [34].

### Measurement of IL-8

HEK293A cell culture supernatants were collected after 20hstimulation, and the IL-8 concentration was determined by a capture enzyme-linked immunosorbent assay (ELISA) using the OptEIA Human IL-8 ELISA Kit (BD Biosciences), in accordance with the manufacturer's instructions, except that signal was detected using a chemiluminescent substrate (SuperSignal West Pico, Pierce). Recombinant human IL-8 provided in the kit was used to generate standard curves.

### Quantitative reverse transcription (RT) PCR for detection of TLR2 transcripts on macrophages

Peritoneal macrophages (2×10^6^ cells/mL) from C57BL/6 mice were distributed in 24-well microplates and incubated at 37°C in a humidified atmosphere of 5% CO_2_. RNA from macrophages stimulated for 5 h with ArtinM (39 nM) was isolated using the TRIzol Reagent (Life Technologies, Carlsbad, CA), according to the manufacturer's instruction. The total RNA was reverse-transcribed into cDNA by the ImProm-II Reverse Transcription System (Promega, Fitchburg, WI) using oligo(dT). Quantitative real-time PCR (qRT-PCR) was performed in 10-µL reactions using SYBR Green (Applied Biosystems/Life Technologies, Carlsbad, CA), 1 µL cDNA (1∶5 dilution), and 0.3 µM primer mix. All the reactions were performed on a 7500 Real-Time PCR System (Applied Biosystems) using the following conditions: 50°C for 2 min, 95°C for 10 min, and 40 cycles of 95°C for 15 sec/60°C for 1 min. Gene expression was quantified using the ΔΔCt method [35] and normalized to β-actin expression. PCR primers utilized were: TLR2: F: 5′CTCTGACCCGCCCTTTAAGC, R: 5′TTTTGTGGCTCTTTTCGATGG; β-actin: F: 5′ CCTAAGGCCAACCGTGAAAA, R: 5′ GAGGCATACAGGGACAGCACA.

### ArtinM binding to TLR2

Peritoneal macrophages (2×10^6^ cells/mL) from C57BL/6 mice were fixed (3% formaldehyde-phosphate-buffered saline) and after 2 washes with phosphate-buffered saline (PBS) were incubated with anti-TLR2 antibody or non-specific IgG (20 µg/mL) for 40 min at room temperature. After washing, biotinylated ArtinM (40 µg/mL) was added for 40 min and after 2 washes, bound biotinylated ArtinM on macrophages was assessed with streptavidin-FITC (strp/FITC; 5 µg/mL; Invitrogen). Fluorescence staining was analyzed by flow cytometry (Guava easyCyte, Guava Technologies, Millipore), after two washes with PBS. The results were expressed as the percentage of cells positive for ArtinM binding versus the median fluorescence intensity (MFI).

To evaluate the dependence of ArtinM-TLR2 binding on carbohydrate recognition, a lysate of peritoneal macrophages (from C57BL/6 mice) was prepared using RIPA lysis buffer. The lysate was then added to the microplate wells previously coated with anti-TLR2 antibody (20 µg/mL) (Santa Cruz Biotechnology, Inc.). Following three washes with PBS, biotinylated ArtinM (40 µg/mL), previously incubated with or without Manα1-3[Manα1-6]Man (trimannoside) (1 mM) or Galα(1,6)Galα(1,6)Gluα(1,2)Fru (stachyose) (50 mM), was then added to the wells. The interaction of ArtinM with TLR2 was detected with neutravidin-alkaline phosphatase (AP) (Pierce) after 1-h incubation at room temperature, followed by the addition of the p-nitrophenyl phosphate (Sigma-Aldrich, St. Louis, MO). Optical density values at 450 nm (OD_450_) were obtained from ArtinM binding to microplates in the presence of a carbohydrate that was either specific for ArtinM binding (trimmanoside) or non-specific (stachyose). These values were normalized to OD_450_ values obtained from ArtinM binding to TLR2 in the absence of any kind of carbohydrate competition. Thus, the relative result is a comparison between the sugar-bound ArtinM and free ArtinM. The OD_450_ was measured using a microplate reader (PowerWave_x_, Biotek Instruments Inc.).

### Cytokine production by ArtinM-stimulated macrophages from TLR2KO mice

Adherent spleen cells or peritoneal macrophages from C57BL/6 (WT) or TLR2 knockout (KO) mice were used to assess IL-12p40 and IL-10 production in response to stimulation with ArtinM. Spleen cells (1×10^7^/mL) were distributed in a 24-well microplate and cultured at 37°Cfor 2 h in a humidified atmosphere with 5% CO_2_. Cells were washed with RPMI 1640 and incubated with ArtinM (156 nM) or Pam3CSK4 (P3C4, 1 µg/mL) (Invivogen) for 24 h. Peritoneal macrophages (2×10^6^/mL) from WT and TLR2-KO mice were also assayed. Cells were plated in a 96-well microplate and cultivated for 12 h under conditions similar to those adopted for adherent spleen cells. Peritoneal macrophages were incubated for 48 h with ArtinM (39 nM) or P3C4 (1 µg/mL). The amount of IL-12 p40 and IL-10 in the culture supernatants was measured by ELISA, using specific antibody pairs purchased from Pharmingen (San Diego, CA) in accordance with the manufacture's protocol. Murine recombinant cytokines were used to create standard curves. IL-12 p40 and IL-10 cytokine concentrations were determined with reference to the standard curves.

### Effect of cell signaling inhibitors on the ArtinM-induced activation of macrophages

Peritoneal macrophages (2×10^6^/mL) from C57BL/6 mice, distributed in 96-well microplates, were pretreated for 3 h in RPMI 1640 with 10% FCS at 37°C in a humidified atmosphere of 5% CO_2_ with the following pharmacological inhibitors: PD98059 (p42/44 mitogen-associated protein kinase [MAPK] inhibitor, 20 µM), SB202190 (p38 MAPK inhibitor, 20 µM), SP600125 (c-Jun N-terminal kinase inhibitor, 25 µM), and LY-294002 (PI3K inhibitor, 20 µM) (Sigma-Aldrich, St. Louis, MO). Subsequently, the peritoneal macrophages were stimulated with ArtinM (39 nM) for 48 h. IL-12p40 and IL-10 concentrations (pg/mL) in the culture supernatants were determined by ELISA, as described in the previous section.

### Statistical analysis

All data were analyzed using Prism (Graph Pad Software). Results are presented as the mean ± standard error of the mean(SEM). Statistical differences were assessed by one-way analysis of variance followed by Bonferroni's multiple comparison test. Differences with p<0.05 were considered statistically significant.

## Results

### ArtinM interaction with TLR2

**Figure 1:**
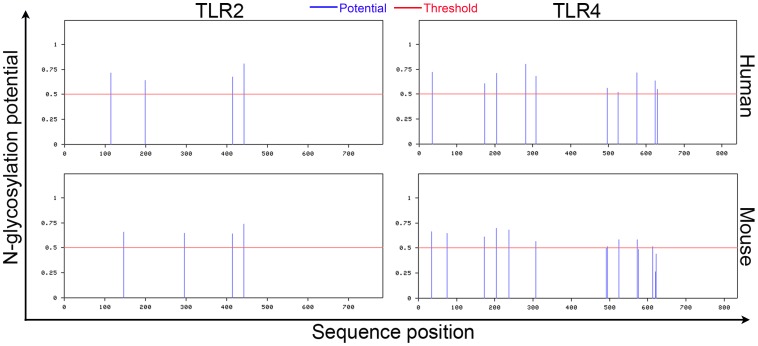
Prediction of N-glycosylation sites in human and murine TLR2 and TLR4. The amino acids sequences of the receptors TLR2 and TLR4 were uploaded to the NetNGlyc 1.0 Server (http://www.cbs.dtu.dk/services/NetNGlyc/) and submitted for analysis. Potential sites for N-glycosylation are indicated by lines (blue) above the threshold (red line). The x-axis indicates the position in the protein sequence of potential glycosylation sites.

TLR2 is directly involved in the protection against *P. brasiliensis* infection conferred by ArtinM administration [19], and TLR4 plays an important role in the recognition of fungi [36]. Therefore, we selected TLR2 and TLR4 as possible targets for ArtinM recognition. The sequences of TLR2 and TLR4 were examined for potential N-glycosylation sites (Figure 1). Four N-glycosylation sites were predicted in human and murine TLR2; two of them(Asn414 and Asn442) similarly positioned in both protein sequences. A previous study demonstrated that all four predicted sites are occupied by N-glycans, which are involved in receptor synthesis, secretion, and function (Weber, Morse *et al*., 2004). Numerous N-glycosylation sites were also predicted in TLR4 but none were positioned similarly in the human and murine proteins (Figure 1).

**Figure 2:**
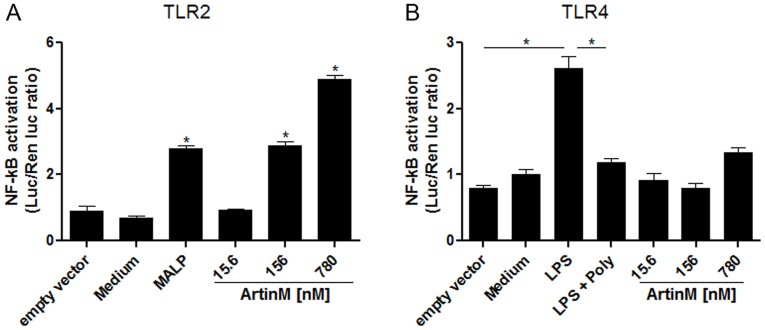
ArtinM triggers TLR2-mediated cell activation. HEK293A cells were transfected with the ectodomain of human TLR2 (A) or human TLR4 (B), and the necessary co-receptors (CD14, CD36, and MD-2), as well as an NF-κB reporter construct and a *Renilla* luciferase control reporter plasmid. The cells were then stimulated with different concentrations of ArtinM (15.6, 156, and 780 nM) at 37°C for 18 h. In (A), MALP-2 (50 ng/mL) was used as the positive control. In (B), LPS (1 µg/mL) was used as the positive control; the addition of polymyxin B (100 µg/mL) to LPS served as another control. In both A and B, medium and an empty vector were used as the negative controls. The luciferase activity was measured as described in materials and methods. Statistical comparisons between unstimulated and stimulated cells were performed using a one-way analysis of variance followed by Bonferroni's multiple comparison test. * p<0.05.

We next assessed the ArtinM interaction with TLRs on the cell surface. HEK293A cells were transfected with plasmids encoding the ectodomain of TLR2 or TLR4; the co-receptors CD14, CD36, and MD-2;and an NF-κB-dependent ELAM-luciferase reporter gene construct. We detected cell activation using a luciferase assay for NF-κB, which showed that ArtinM interacted with TLR2 in a dose-dependent manner. The activation level in cells stimulated with 156 nM ArtinM was similar to that in cells stimulated with the control agonist MALP2. These activation levels were markedly different from those obtained with the negative controls(medium or cells transfected with an empty vector) (Figure 2A). In contrast, ArtinM did not induce NF-κB activation in HEK293A cells expressing TLR4, although the cells were fully responsive to stimulation with LPS (positive control) (Figure 2B). These data demonstrated that ArtinM acted as a TLR2 agonist to induce an NF-κB response.

### ArtinM interaction with TLR2 heterodimers

**Figure 3:**
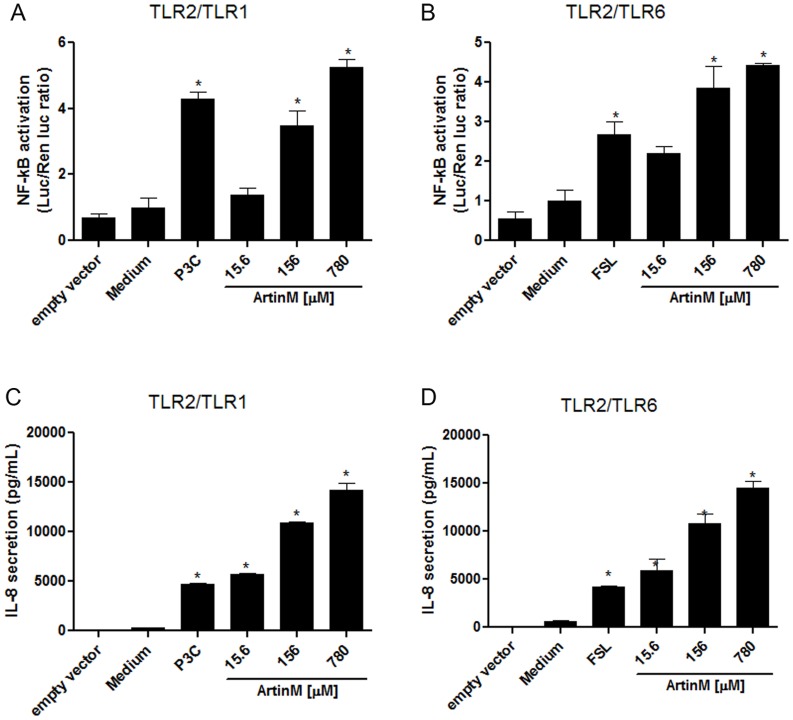
ArtinM induces the activation of TLR2/1- and TLR2/6-expressing cells. HEK293A cells were transfected with TLR2/1 (A and C) or TLR2/6 (B and D), co-receptors, an NF-κB reporter construct, and a *Renilla* luciferase reporter plasmid as described for Figure 2. The transfected cells were stimulated with ArtinM (15.6, 156, and 780 nM) at 37°C for 4 h. Medium and cells transfected with an empty vector were used as the negative controls. The positive controls were P3C4 (1 nM) for TLR2/1 activation (A and C) and FSL1 (0.1 nM) for TLR2/6 activation (B and D). The luciferase activity (A and B) was measured as described in the materials and methods. IL-8 levels in the culture supernatants (C and D) were measured by ELISA. Statistical comparisons between the cells incubated with medium and the cells stimulated with ArtinM were performed with a one-way analysis of variance followed by Bonferroni's multiple comparison test. * p<0.05.

Because TLR2 forms heterodimers with TLR1 orTLR6 on the cell surface, we investigated whether HEK293A cells transfected with TLR2/1 or TLR2/6, as well as with the accessory proteins CD16 and CD36, interacted with ArtinM. As described above, the cells were also transfected with the NF-κB-dependent ELAM-luciferase reporter gene construct, whose activation was detected with a luciferase assay. Alternatively, cell activation was assessed by measuring the concentration of IL-8 in the culture supernatants. The luciferase assay (Figure 3A and B) and the IL-8 measurements (Figure 3C and D) showed that ArtinM interacted with both TLR2/1 and TLR2/6 in a dose-dependent manner. The activation levels in cells stimulated with 156 nM ArtinM were similar to those in cells treated with the control agonists P3C4 (TLR2/1) and FSL (TLR2/6), but markedly different from those obtained with the negative controls (medium or cells transfected with an empty vector). Measurement of NF-κB activation and IL-8 release yielded consistent results and provided functional evidence that ArtinM targets macromolecular complexes containing TLR2.

### ArtinM increases TLR2 relative expression in peritoneal macrophages

**Figure 4:**
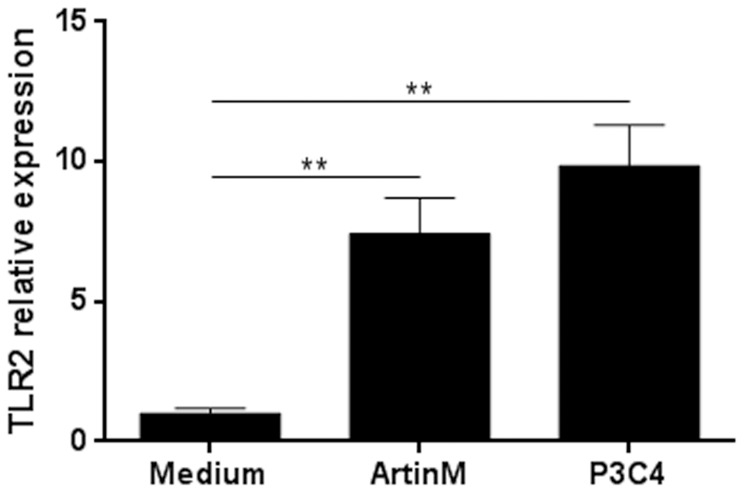
Enhanced TLR2 relative expression by ArtinM-stimulated macrophages. Peritoneal macrophages from C57BL/6 mice were incubated with ArtinM (39 nM) for 5 h. Medium was used as a negative control and P3C4 (1 µg/mL) was used as a positive control. RNA from macrophages were isolated and used for qRT-PCR as described in Materials and Methods. The results are expressed as the relative expression of TLR2 after quantification using the ΔΔCt method and normalized to β-actin expression. Statistical comparisons between stimulated cells and unstimulated were performed with one-way analysis of variance followed by Bonferroni's multiple comparison test. ** p<0.01.

Having demonstrated the ArtinM interacted with TLR2 in HEK293A cells, we next investigated the effects of ArtinM on murine macrophages, specifically the ability of ArtinM to enhance the expression of TLR2 mRNA. Thioglycollate-elicited peritoneal macrophages were harvested from C57BL/6 mice and stimulated with ArtinM (39 nM) for 5 h. Quantitative analysis of TLR2 mRNA showed that the relative expression of TLR2 increased in ArtinM-stimulated macrophages. Moreover, stimulation with P3C4 increased the TLR2 relative expression (Figure 4). Thus, in addition to interact with TLR2, ArtinM enhanced the expression of TLR2 mRNA on macrophages.

### ArtinM interacts with TLR2 in a sugar recognition-dependent manner

**Figure 5:**
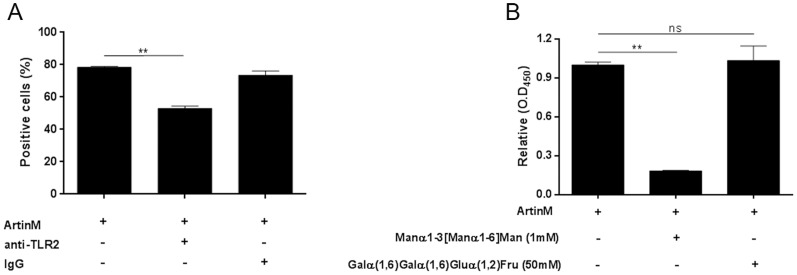
ArtinM binding to TLR2 depends on sugar recognition. (A and B) Peritoneal macrophages from C57BL/6 mice were incubated with biotinylated ArtinM after pre-incubation with anti-TLR2 antibody or non-specific IgG. ArtinM binding was detected with streptavidin-FITC and analyzed by flow cytometry, as described in materials and methods. Results are expressed as the percentage of cells positive for ArtinM binding (A) and MFI (median fluorescence intensity) (B). (C) The dependence of ArtinM-TLR2 binding on carbohydrate recognition used anti-TLR2 antibody coated onto 96-well microplates (5 µg/mL) to capture TLR2 from a cellular extract of peritoneal macrophages. Biotinylated ArtinM (40 µg/mL), previously incubated with the indicated concentrations of Manα1-3 [Manα1-6] Man or Galα(1,6)Galα(1,6)Gluα(1,2)Fru, was added to the wells. After washing, ArtinM binding was detected by neutravidin-AP, and signal was developed with *p*-nitrophenyl phosphate. Results are expressed in O.D as the mean ± SEM. Statistical comparisons between cells incubated or not with carbohydrates were performed with one-way analysis of variance followed by Bonferroni's multiple comparison test. *p<0.05.

To further characterize the interaction of ArtinM with TLR2, we evaluated the ability of an anti-TLR2 antibody to inhibit ArtinM binding to the surface of murine macrophages. Cells harvested from the peritoneal cavity of C57BL/6 mice were pre-incubated with anti-TLR2 antibody. Then, they were washed, incubated with biotinylated ArtinM, and reacted with a streptavidin-FITC conjugate. Flow cytometric analysis showed that pre-incubation of cells with anti-TLR2 antibody decreased the number of ArtinM-labeled cells by 30% (Figure 5A) and the median fluorescence intensity reduced 71.5% (Figure 5B), suggesting that TLR2 is a major ligand for ArtinM on the surface of murine macrophages.

To investigate whether ArtinM targets TLR2 N-glycans on the surface of murine macrophages, we performed a sugar-inhibition assay using TLR2 molecules that were captured from an extract of peritoneal macrophages by a specific antibody. ArtinM binding to TLR2 was strongly inhibited by pre-incubation of ArtinM with Manα1-3[Manα1-6]Man, but was not affected by pre-incubation of ArtinM with a non-specific galactose-containing trisaccharide, stachyose (Figure 5C). Therefore, ArtinM recognition of the trimannoside core of N-glycans is critical for its interaction with TLR2.

### TLR2 mediates the activation of macrophages induced by ArtinM

**Figure 6:**
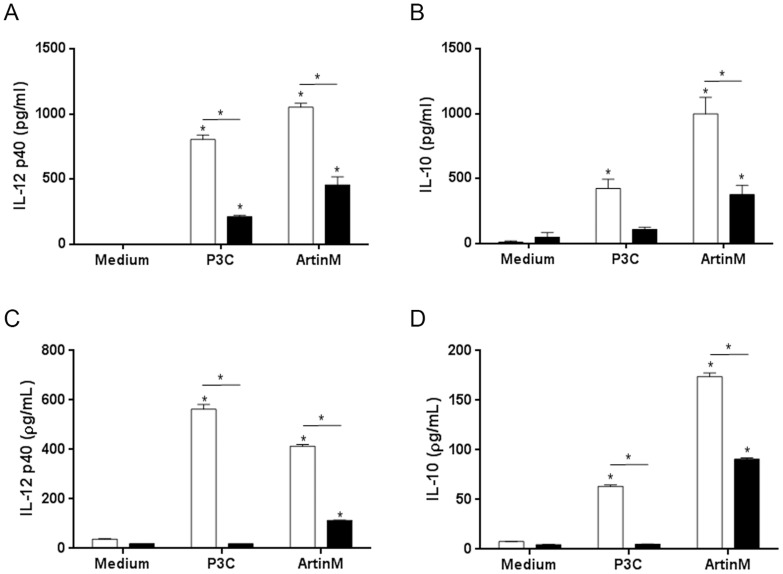
TLR2 mediates the cytokine production induced by ArtinM. IL-12p40 and IL-10 levels in the cell culture supernatants of adherent spleen cells (A and B) or peritoneal macrophages (C and D) from WT (white bars) and TLR2-KO (black bars) mice were measured by ELISA. Adherent spleen cells were stimulated with ArtinM (156 nM) or P3C4 (1 µg/mL) for 24 h, while the peritoneal macrophages were incubated with ArtinM (39 nM) or P3C4 (1 µg/mL) for 48 h. Statistical comparisons between unstimulated and stimulated cells were performed using a one-way analysis of variance followed by Bonferroni's multiple comparison test. *p<0.05.

To investigate the biological relevance of the interaction of ArtinM with TLR2, we compared cytokine production by macrophages fromTLR2-KO and WT mice after stimulation with ArtinM for 24 h. Cell suspensions of peritoneal macrophages or adherent spleen cells derived from TLR2-KO mice produced significantly lower levels of IL-12p40 (41%) and IL-10 (42%) than those obtained from WT mice, in response to ArtinM stimulation (Figure 6). These results indicate that the ArtinM-induced production of cytokines requires the TLR2signaling pathway. Although production of IL-12 and IL-10 was still detected in cells from TLR2KO mice after stimulation with ArtinM, cytokine production was not detected after stimulation with the control TLR2 agonist P3C4. This result suggests that, in addition to TLR2, whose biological relevance is indubitable, additional(s) receptor(s) may contribute to the cell signaling and cytokine production triggered by ArtinM.

### Signaling molecules involved in ArtinM-induced macrophage activation

**Figure 7:**
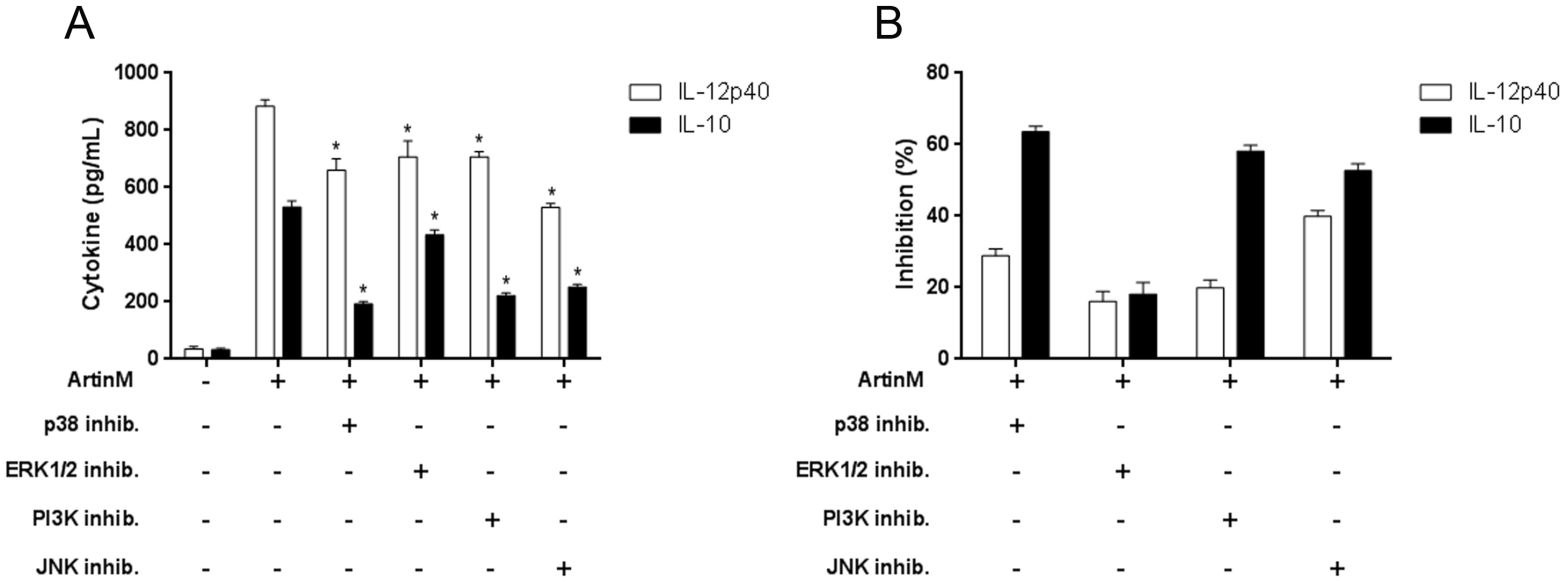
Cell signaling molecules that possibly mediate the macrophage activation induced by ArtinM. Peritoneal macrophages were pretreated with the inhibitors PD98059 (p42/44 MAPK or ERK 1/2), SB202190 (p38 MAPK), SP600125 (c-Jun N-terminal kinase), and LY-294002 (PI3K), or with medium alone (absence of inhibitor) and then stimulated with ArtinM (39 nM). After 48 h, IL-12p40 and IL-10 levels in the culture supernatants were assessed by ELISA. (A) Cytokine production reported as pg/mL (mean ± SEM) and statistical comparison were performed using a one-way analysis of variance followed by Bonferroni's multiple comparison test. *p<0.05. (B) Inhibition of ArtinM induced cytokine production after pre-incubation with the indicated inhibitors. The results are presented as the percent inhibition, which represents the ratio between uninhibited cells and those pretreated with inhibitors.

Because full activation of macrophages by ArtinM requires interaction with TLR2, we investigated the signaling pathways used to promote IL-12p40 and IL-10 production by assessing the effects of several pharmacological inhibitors (PD98059, SB202190, SP600125, and LY-294002) on the response to ArtinM stimulation. We evaluated IL-12p40 and IL-10 production by macrophages that were treated with inhibitors and then stimulated with ArtinM. IL-12p40 production induced by ArtinM was significantly inhibited by SB202190 and SP600125, indicating the involvement of p38 MAPK and JNK (Figure 7). In addition to these signaling molecules, PI3K was involved in IL-10 production, as indicated by the inhibitory effect of LY-294002 (Figure 7). Therefore, the signaling molecules activated in ArtinM-stimulated macrophages are partially specific for the production of different cytokines.

## Discussion

The immunomodulatory activity of ArtinM was previously demonstrated by studies in which lectin administration induced Th1 immunity in *P. brasiliensis*-inoculated mice and conferred protection against infection in a TLR2-dependent manner [19]. However, a direct physical interaction ArtinM with TLR2 has not been demonstrated. In the present study, by using murine macrophages and HEK293A cells transfected with plasmids expressing the ectodomain of TLR2, we demonstrated that ArtinM interacted with TLR2 via carbohydrate recognition.

TLRs, which recognize conserved pathogen motifs and initiate an inflammatory signaling cascade, play essential roles in host defense against a variety of pathogens, including some that cause pulmonary infections [37], [38]. Because the protection conferred by ArtinM against *P. brasiliensis* infection depends on TLR2 activation and some lectins were previously reported to function as TLR agonists [25], we investigated if ArtinM interacted directly with TLR2. Studies showing that TLR2 needs to heterodimerize with TLR1 or TLR6 to initiate signaling and cellular activation [6], [39], [40] led us to assess the ability of ArtinM to activate HEK293A cells transfected with the TLR2 ectodomain alone, TLR2/1, TLR2/6, or TLR4. Cells transfected with TLR2, but not with TLR4, responded to ArtinM. Consistent with this result, anti-TLR2 specific antibodies reduced ArtinM binding to murine macrophages by more than 30%. Thus, we demonstrated that TLR2 is an important target of ArtinM.

Our findings and results in the literature concerning the detection of four N-glycans attached to the TLR2 ectodomain [41], one of which (at Asn442) is essential for the recognition of TLR2 agonists [42], motivated us to investigate if ArtinM carbohydrate recognition domains (CRDs) were responsible for the binding to TLR2. The trimannoside Manα1-3[Manα1-6]Man selectively blocked ArtinM binding to TLR2, indicating that the interaction is dependent on sugar recognition. Although we did not identify which TLR2 N-glycan(s) accounted for the cell activation triggered by ArtinM, we consider Asn442 a good candidate for future investigations.

Stimulation of murine macrophages with ArtinM also enhanced TLR2 expression on the cell surface, a result that is compatible with the finding that Concanavalin A (Con A) induces higher TLR2–9 expression in murine peritoneal macrophages [43], [44]. TLR2 expression is differentially regulated by inflammatory mediators [45]–[48], and some infections have been associated with increased TLR2 expression [49]–[54]. Thus, in macrophages stimulated with ArtinM, higher levels of TLR2 may create an additional activation loop that promotes cell response to new lectin molecules and to inflammatory mediators secreted *de novo*.

Recently, tunicamycin, a known inhibitor of N-linked glycoprotein synthesis, was reported to suppress the TLR-mediated induction of proinflammatory molecules. The effect was associated with decreased NF-κB. The anti-inflammatory effect of tunicamycin was attributed, at least in part, to the inhibition of *N*-glycosylation on cell receptors [55] such as TLRs. TLR2 binding to agonists is impaired by the absence of the *N*-glycan linked to Asn442 in the receptor ectodomain [42]. In the present study, we found that macrophage activation was induced by the interaction of the ArtinM CRD with *N*-glycans linked to TLR2 molecules on the cell surface. TLR2 participation was supported by the marked reduction in ArtinM-induced cytokine production in TLR2-/- mice, in peritoneal macrophages and adherent spleen cells, which may have distinct N-glycans on their surface. Notably, the TLR2-mediated response to ArtinM involved not only the production of IL-12, an inflammatory cytokine that is crucial for the development of Th1 immunity, but also the production of IL-10, which may play an anti-inflammatory role that contributes to immune homeostasis. The augmented release of IL-10 found in this study was not associated with the production of other Th2 cytokines.

The decrease in cytokine production after treatment with inhibitors of signaling molecules suggested that ArtinM acted mainly through p38 MAPK, PI3K, and JNK to stimulate IL-10 production and through p38 MAPK and JNK to stimulate IL-12p40 production. Nonetheless, the involvement of these molecules in ArtinM-induced cytokine production requires further analysis and verification. It was previously reported that peritoneal macrophages stimulated with wheat germ agglutinin (WGA) produce proinflammatory cytokines such as TNF-α, IL-1β, IL-12, and IFN-γ in a manner that is dose- and time-dependent, as well as sensitive to different pharmacological inhibitors (p38 MAPK, PI3K, JNK, and ERK1/2). In addition, IL-1β production by macrophages stimulated with ConA occurs through the PI3K, PKC, ERK 1/2, p38 MAPK, and JAK/STAT pathways [43]. These data suggest that lectins with different specificities may induce macrophage activation through several signaling pathways by targeting different glycosylated receptors.

In summary, our study showed that ArtinM interacted with TLR2 and its heterodimers in a carbohydrate recognition-dependent manner. The cytokine profile induced by this interaction was consistent with the ability of ArtinM to control infections by intracellular pathogens and prevent severe immunopathology. The present work provides a new perspective on a novel TLR agonist that acts through carbohydrate recognition to modulate the immune response against different pathogens.
